# Adaptive Processing of Multi-Source Drilling Sensor Signals for Coal–Rock Identification Under Limited Drilling Data: A Laboratory Case Study

**DOI:** 10.3390/s26206607

**Published:** 2026-10-19

**Authors:** Bin Liang, Dong Wei, Yurou Wang, Yongyuan Ma

**Affiliations:** 1 School of Mechatronic Engineering, China University of Mining and Technology, No. 1 Daxue Road, Xuzhou 221116, China; lbxhxy@163.com (B.L.); ts24050214p31@cumt.edu.cn (Y.W.); ts24050041a31ld@cumt.edu.cn (Y.M.); 2 Xu Hai College, China University of Mining and Technology, No. 1 Jindong Road, Xuzhou 221008, China

**Keywords:** drilling monitoring, multi-source sensors, coal–rock identification, variational mode decomposition, wavelet thresholding, limited drilling data, sensor signal processing

## Abstract

Reliable coal–rock identification during drilling is challenged by limited labeled data and non-stationary, strongly coupled multi-source signals. This laboratory case study evaluates a recognition-oriented adaptive framework, IMGO–VMD–WT, that uses torque-guided improved moss growth optimization (IMGO) to select variational mode decomposition (VMD) parameters, grades intrinsic mode functions by information content, applies differentiated wavelet-threshold processing, and reconstructs the signal with residual preservation. A self-built laboratory drilling rig provided data from six material categories and eleven pressure, displacement, torque, and rotational-speed channels. Under five-fold contiguous-block validation, raw features, wavelet thresholding, fixed VMD–WT, PSO–VMD–WT, GWO–VMD–WT, and IMGO–VMD–WT achieved 90.26%, 91.94%, 92.17%, 92.13%, 92.13%, and 92.57% accuracy, respectively, with corresponding macro-F1 scores of 78.34%, 80.37%, 81.73%, 82.17%, 82.17%, and 82.22%. The exploratory paired comparison between IMGO–VMD–WT and fixed VMD–WT gave accuracy and macro-F1 differences of 0.40 and 0.49 percentage points, with exact sign-permutation *p*-values of 0.6875 and 0.5625 and bootstrap 95% intervals that included zero. In a controlled 10 dB noise-injection test on 12 measured torque windows, IMGO–VMD–WT reached 19.19 dB output SNR and an RMSE of 1.379. These results support decomposition-aware processing as a feasible offline strategy for limited-data laboratory drilling analysis.

## 1. Introduction

Coal–rock identification during drilling provides direct information about the material being cut, the interaction between the bit and the surrounding medium, and the operating state of the drilling mechanism. It is therefore relevant to intelligent drilling control, roof and roadway support, outburst prevention, and automated mining equipment. Compared with manual observation or post-drilling core inspection, sensor-based identification can provide continuous and low-latency information during the drilling process. Pressure, displacement, torque, and rotational-speed sensors are particularly attractive because they can be integrated into hydraulic drilling systems and reflect load transfer, cutting resistance, feed motion, and speed regulation [1,2,3]. They are of great significance for the intelligent control and operation of the drilling robots for preventing impact ground pressure.

Recent studies have connected drilling-process monitoring with tool condition, rock-mass assessment, and data-driven signal analysis. Landar et al. examined drilling-tool vibration metrics for bit wear and penetration-rate decline [4]; Mussin et al. combined geophysical measurements to characterize structural weakening in coal masses [5]; and Rysbekov et al. discussed integrated monitoring of rock-mass state and mining safety [6]. Ahmed and Nandi demonstrated the use of compressed vibration measurements, learned features, and support-vector classification for condition monitoring [7]. These studies broaden the international context, whereas the present work focuses on synchronized pressure, displacement, torque, and rotational-speed signals for laboratory coal– rock identification.

For this problem, the main difficulty is that drilling signals are not clean responses of rock breakage alone. They contain hydraulic-pump pulsation, valve dynamics, motor fluctuation, transmission vibration, sensor drift, bit–rock impact, and short-time disturbances. Acoustic drilling responses can also carry lithological information, but they are strongly affected by propagation and acquisition conditions [8]. These sources are coupled in a nonlinear and non-stationary manner. Moreover, laboratory drilling datasets are often limited because preparing coal–rock samples, repeating drilling tests, and maintaining identical operating conditions is costly. Under such conditions, directly training a classifier on raw time series can be problematic: apparent high accuracy may come from data leakage between adjacent windows, while weak generalization may occur when the same method is applied to another coal–rock block. This issue is consistent with broader time-series classification studies, where validation design and dataset independence strongly influence reported performance [9,10].

Signal decomposition and adaptive denoising provide a practical route for limited-data sensor learning. Variational mode decomposition (VMD) decomposes a non-stationary signal into band-limited intrinsic mode functions (IMFs), reducing mode mixing compared with empirical sifting methods and allowing the signal to be described at multiple frequency scales [11,12,13]. However, VMD is sensitive to the mode number *K* and the penalty factor α. If *K* is too small, mechanically different components may be mixed. If *K* is too large, over-decomposition introduces redundant modes and weakens feature stability. The penalty factor also affects the bandwidth constraint and the preservation of transient drilling impacts. Manual parameter selection is therefore unsuitable for heterogeneous drilling data.

Wavelet-threshold denoising is another well-established tool for non-stationary signals because it suppresses small coefficients while preserving localized transients [14,15,16]. Hybrid VMD–WT strategies have been used in hydrophone, acoustic-emission, and transient-electromagnetic denoising tasks, often using optimized VMD parameters and mode-wise thresholding [17,18,19]. Nevertheless, applying the same threshold rule to all frequency components is not ideal for drilling signals. High-confidence modes that represent cutting load and impact should not be over-smoothed, whereas noise-dominant modes should be strongly suppressed. This motivates a decomposition-aware denoising strategy that treats different IMFs differently.

This study develops an adaptive multi-source sensor signal-processing framework for coal–rock identification under limited drilling data. The method uses improved moss growth optimization (IMGO), developed from moss growth optimization and related population-search principles [20,21,22,23], to select VMD parameters for each drilling window. It then grades IMFs according to their information content and applies differentiated wavelet-threshold operations before reconstructing the processed signal with an original-signal preservation term. The core idea is recognition-oriented reconstruction: the decomposition is not only used to suppress noise, but also to retain the load and motion structure that is most useful for downstream coal–rock classification.

The contributions are as follows:

1. An improved moss growth optimization algorithm is developed for adaptive VMD parameter selection. By integrating diversified initialization, representative anchor candidates, elite-guided search, adaptive diffusion and reproduction, and perturbation-based exploration, the proposed IMGO searches for the mode number *K* and penalty factor α using a decomposition-quality fitness function, thereby reducing the dependence of VMD on manually selected parameters.
2. A coal–rock identification method based on the proposed optimization algorithm is constructed. In this method, IMGO provides window-level VMD parameters guided by the torque channel, the decomposed IMFs are graded according to information content, differentiated wavelet-threshold processing and residual-preserving reconstruction are performed, and features extracted from eleven pressure, displacement, torque, and rotational-speed channels are used for coal–rock classification.
3. The implementation performance of the proposed method is verified on a self-built drilling test platform. For 245 one-second windows derived from 247,680 labeled sensor sampling points from six drilling material categories, the proposed IMGO–VMD–WT method achieved 92.57% accuracy and 82.22% macro-F1 under five-fold contiguous-block validation. In the controlled 10 dB noise-injection test, the method reached 19.19 dB output SNR and an RMSE of 1.379.

## 2. Proposed Method

This section presents the proposed adaptive signal-processing framework for coal–rock identification from multi-source drilling sensor signals. The framework first divides the measured signals into one-second windows and uses the torque channel to guide adaptive VMD parameter selection through the improved moss growth optimization algorithm. The resulting intrinsic mode functions are then evaluated according to their information content, processed using differentiated wavelet-threshold rules, and recombined with a residual-preserving strategy. Finally, features extracted from the eleven synchronized sensor channels are used for coal–rock classification. The following subsections describe the processing objective, overall workflow, parameter optimization, IMF scoring, signal reconstruction, and feature extraction procedures.

### 2.1. Processing Objective and Data Unit

Let $x_{c}\left[n\right]$ denote a discrete drilling signal from channel *c*, where c=1,…,C and C=11. The objective is to produce a processed signal ${\hat{x}}_{c}\left[n\right]$ that suppresses noise-dominant components while preserving material-sensitive load, motion, and pressure responses. The measured signals are divided into non-overlapping one-second windows, and all eleven channels are used for feature extraction. In the adaptive method, the torque channel serves as the representative load signal for IMGO parameter selection in each window, and the resulting $\left(K^{*},\alpha^{*}\right)$ is then applied to all channels in that window. This keeps the parameter search aligned with the strongest load-bearing channel while maintaining cross-channel comparability. The final output uses residual-preserving fusion between the VMD–WT reconstruction and the measured signal so that trend-level drilling behavior and transient cutting responses are retained.

### 2.2. Overall Framework

**Figure 1:**
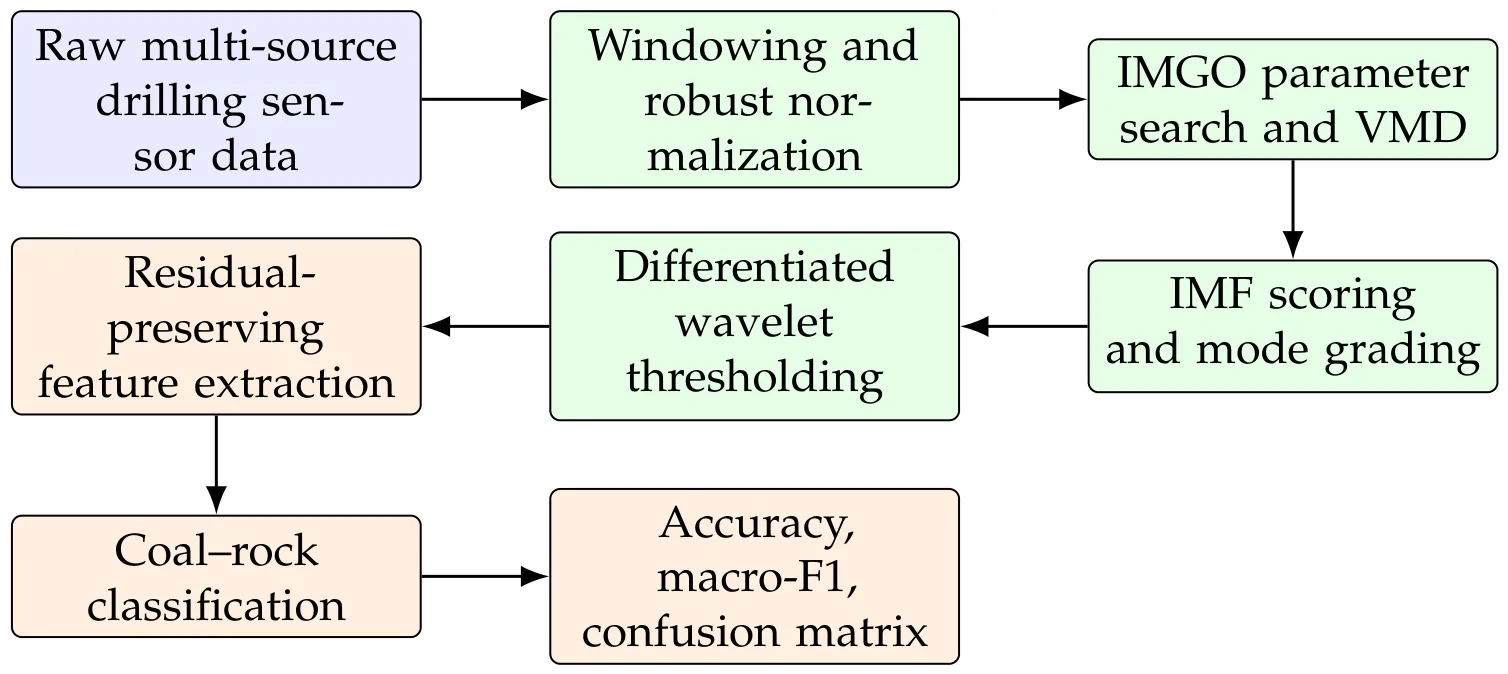
Adaptive multi-source sensor processing framework for limited-data coal–rock identification.

Figure 1 summarizes the proposed adaptive framework. The workflow contains five modules: data segmentation, adaptive decomposition, IMF scoring, differentiated denoising, and coal–rock identification. The method is deliberately modular. This allows the denoising component to be evaluated both as a signal-processing method and as a preprocessing step for classification.

### 2.3. Variational Mode Decomposition

VMD models a signal as the sum of *K* band-limited modes $u_{k}\left(t\right)$ with center frequencies $\omega_{k}$ [11]. For a continuous signal f(t), the constrained variational problem is

(1) $$\min_{\left\{u_{k}\right\},\left\{\omega_{k}\right\}}\left\{\sum_{k=1}^{K}{∥\partial_{t}\left[\left(\delta \left(t\right)+\frac{j}{\pi t}\right)*u_{k}\left(t\right)\right]e^{-j\omega_{k}t}∥}_{2}^{2}\right\},\;s.t.\;\sum_{k=1}^{K}u_{k}\left(t\right)=f\left(t\right).$$

In practical implementation, the problem is solved by the alternating direction method of multipliers. The two most influential parameters are the mode number *K* and the penalty factor α. The present work treats both as adaptive parameters selected by IMGO for each signal window or for each representative segment.

### 2.4. IMGO-Based Parameter Selection

The original moss growth optimization algorithm is a population-based metaheuristic inspired by moss propagation and cryptobiosis [20]. In this study, IMGO is used to select the VMD parameter vector

(2) $$z=\left[K,\alpha \right],\;K\in \left[K_{\min},K_{\max}\right],\;\alpha \in \left[\alpha_{\min},\alpha_{\max}\right],$$

where *K* is rounded to the nearest feasible integer. The IMGO module combines diversified initialization, anchor candidates over representative (K,α) combinations, elite-guided search, adaptive diffusion and reproduction operators, and perturbation-based exploration. This design gives the parameter-selection module broad coverage of the feasible VMD space while retaining a compact implementation suitable for repeated one-window processing. For independent implementation, each candidate is represented by normalized coordinates $q_{i}\in {\left[0,1\right]}^{2}$ and mapped to $\left(K_{i},\alpha_{i}\right)$ by linear scaling, with $K_{i}$ rounded to the nearest feasible integer. The eight-member initial population comprises boundary and intermediate anchor candidates together with Latin-hypercube/opposition exploratory candidates; duplicates are replaced by uniformly sampled feasible candidates. In round *r*, the elite-guided update is

(3) $$q_{i}^{r+1}=\mathrm{clip}\left[q_{i}^{r}+a_{r}u_{i}^{r}⊙\left(q_{best}^{r}-q_{i}^{r}\right)+\sigma_{r}\epsilon_{i}^{r},0,1\right],$$

where $a_{r}=2\left(1-r/\left(R-1\right)\right)$, $\sigma_{r}=0.20\left(1-r/\left(R-1\right)\right)+0.02$, R=8, $u_{i}^{r}∼U{\left(0,1\right)}^{2}$, and $\epsilon_{i}^{r}∼N\left(0,I\right)$. Diffusion and reproduction probabilities are $p_{diff}\left(r\right)=0.80-0.60r/\left(R-1\right)$ and $p_{rep}\left(r\right)=1-p_{diff}\left(r\right)$. For reproduction, two distinct candidates are sampled and $q_{i,rep}=\mathrm{clip}\left[q_{best}^{r}+u⊙\left(q_{j}^{r}-q_{k}^{r}\right),0,1\right]$. Anchors are reevaluated each round and retained when among the eight lowest-fitness candidates. Stagnation is declared when the best objective does not improve by more than $10^{-12}$ for two consecutive rounds. The elite is then perturbed by 0.05tan[π(v−1/2)], with $v∼U{\left(0,1\right)}^{2}$, and accepted only if the objective decreases. Candidates are clipped to the feasible domain, ranked by *J*, and the eight best are carried forward.

For a candidate parameter vector $z_{i}$, VMD is performed and the decomposition quality is evaluated by

(4) $$J\left(z_{i}\right)=0.36{\bar{H}}_{\mathrm{env}}+0.26E_{\mathrm{rec}}+0.18R_{\mathrm{imf}}+0.12P_{\mathrm{conc}}+0.08P_{\mathrm{bnd}},$$

The five terms are defined explicitly as follows. For the Hilbert envelope $e_{m}\left[n\right]=\left|u_{m}\left[n\right]+jH\left\{u_{m}\left[n\right]\right\}\right|$, let $p_{m}\left[n\right]=e_{m}\left[n\right]/\sum_{n}e_{m}\left[n\right]$ and

(5) $$H_{\mathrm{env},m}=-\frac{\sum_{n}p_{m}\left[n\right]\log\left(p_{m}\left[n\right]+10^{-12}\right)}{\log L},\;{\bar{H}}_{\mathrm{env}}=\frac{1}{K}\sum_{m=1}^{K}H_{\mathrm{env},m},$$

where *L* is the window length. The reconstruction error, average pairwise IMF correlation, maximum energy concentration, and boundary penalty are

(6) $$E_{\mathrm{rec}}=\frac{{∥f-\sum_{m=1}^{K}u_{m}∥}_{2}}{{∥f∥}_{2}},\;R_{\mathrm{imf}}=\frac{2}{K(K-1)}\sum_{r<s}\left|\mathrm{corr}\left(u_{r},u_{s}\right)\right|,$$

(7) $$P_{\mathrm{conc}}=\max_{m}\eta_{m},\;P_{\mathrm{bnd}}=\frac{1}{2}\left(\left|2\frac{K-K_{\min}}{K_{\max}-K_{\min}}-1\right|+\left|2\frac{\alpha -\alpha_{\min}}{\alpha_{\max}-\alpha_{\min}}-1\right|\right).$$

The coefficients (0.36,0.26,0.18,0.12,0.08) are the recommended values obtained through multiple trials on the experimental platform. These values can be pre-adjusted in actual engineering based on the characteristics of different equipment. In the research of this article, the coefficients were fixed before fold evaluation and were not tuned on the test folds. Candidate vectors were compared using the composite decomposition objective and reconstructed-signal stability. The screening was completed before outer five-fold evaluation, and held-out folds were not used. The selected vector was fixed for every method and fold. A smaller *J* indicates lower envelope complexity, lower reconstruction distortion, lower inter-mode redundancy, less single-mode concentration, and a less boundary-dominated parameter choice. The search interval was K∈[3,6] and α∈[400,4000]. The implementation used a population of 8 candidates and 8 iterations per window. Initialization combined Latin-hypercube sampling and opposition candidates. Representative anchor points were retained, the elite direction was used for local movement, diffusion/reproduction probabilities were adapted by iteration, and a Cauchy perturbation was applied after stagnation. The torque window was the only signal optimized, and the selected $\left(K^{*},\alpha^{*}\right)$ was then applied to all eleven channels to reduce computation and keep cross-channel processing comparable. On the analysis computer, the timing assessment gave an average IMGO search time of 28.60±7.90 s per window. Subsequent VMD–WT processing of all eleven channels required 3.40±0.70 s, feature extraction required 0.10±0.02 s, and the complete processing chain required 32.10±8.20 s per window. The selected parameters are

(8) $$\left(K^{*},\alpha^{*}\right)=\arg\min_{z\in \Omega }J\left(z\right).$$

### 2.5. IMF Scoring and Differentiated Wavelet Thresholding

After decomposition with $\left(K^{*},\alpha^{*}\right)$, the *m*-th IMF was evaluated by three indicators:

(9) $$\eta_{m}=\frac{\sum_{n}u_{m}^{2}\left[n\right]}{\sum_{r=1}^{K^{*}}\sum_{n}u_{r}^{2}\left[n\right]},$$

(10) $$\rho_{m}=\left|\frac{\sum_{n}\left(u_{m}\left[n\right]-{\bar{u}}_{m}\right)\left(x\left[n\right]-\bar{x}\right)}{\sqrt{\sum_{n}{\left(u_{m}\left[n\right]-{\bar{u}}_{m}\right)}^{2}}\sqrt{\sum_{n}{\left(x\left[n\right]-\bar{x}\right)}^{2}}}\right|,$$

and the normalized envelope entropy $H_{m}=H_{\mathrm{env},m}$ from Equation (5). The information score was

(11) $$S_{m}=0.45\eta_{m}+0.35\rho_{m}+0.20\left(1-H_{m}\right).$$

The weights in Equation (11) were set to (0.45,0.35,0.20) before fold evaluation to give the energy contribution the largest weight, followed by correlation and then complexity. The grading rule was $S_{m}\geq \theta_{\mathrm{high}}=0.60$ for signal-dominant modes, $0.30\leq S_{m}<0.60$ for mixed modes, and $S_{m}<\theta_{\mathrm{low}}=0.30$ for noise-dominant modes. Signal-dominant modes were retained or weakly thresholded, mixed modes were soft-thresholded, and noise-dominant modes were strongly thresholded or omitted.

The wavelet-thresholding configuration used Daubechies-4 (‘db4’), four decomposition levels, and soft thresholding. The same db4 wavelet, four-level decomposition, scale-wise MAD estimation, universal threshold, and soft-thresholding rule were used for WT, fixed VMD–WT, PSO–VMD–WT, GWO–VMD–WT, and IMGO–VMD–WT. With detail coefficients $d_{j,l}$, $N_{j}$ coefficients at scale *j*, and ${\hat{\sigma }}_{j}=\mathrm{median}(|d_{j,l}\left|\right)/0.6745$, the threshold was $\lambda_{j}={\hat{\sigma }}_{j}\sqrt{2\log N_{j}}$ and

(12) $${\tilde{d}}_{j,l}=\mathrm{sgn}\left(d_{j,l}\right)\max\left(|d_{j,l}|-\lambda_{j},0\right).$$

The VMD–WT signal was reconstructed by summing retained and denoised IMFs:

(13) $${\hat{x}}_{\mathrm{VMD}-\mathrm{WT}}\left[n\right]=\sum_{m\in S}u_{m}\left[n\right]+\sum_{m\in M}{\tilde{u}}_{m}\left[n\right].$$

The final output further preserved the measured drilling trend by

(14) $$\hat{x}\left[n\right]=\beta {\hat{x}}_{\mathrm{VMD}-\mathrm{WT}}\left[n\right]+\left(1-\beta \right)x\left[n\right],\;\beta =0.59.$$

**Figure 2:**
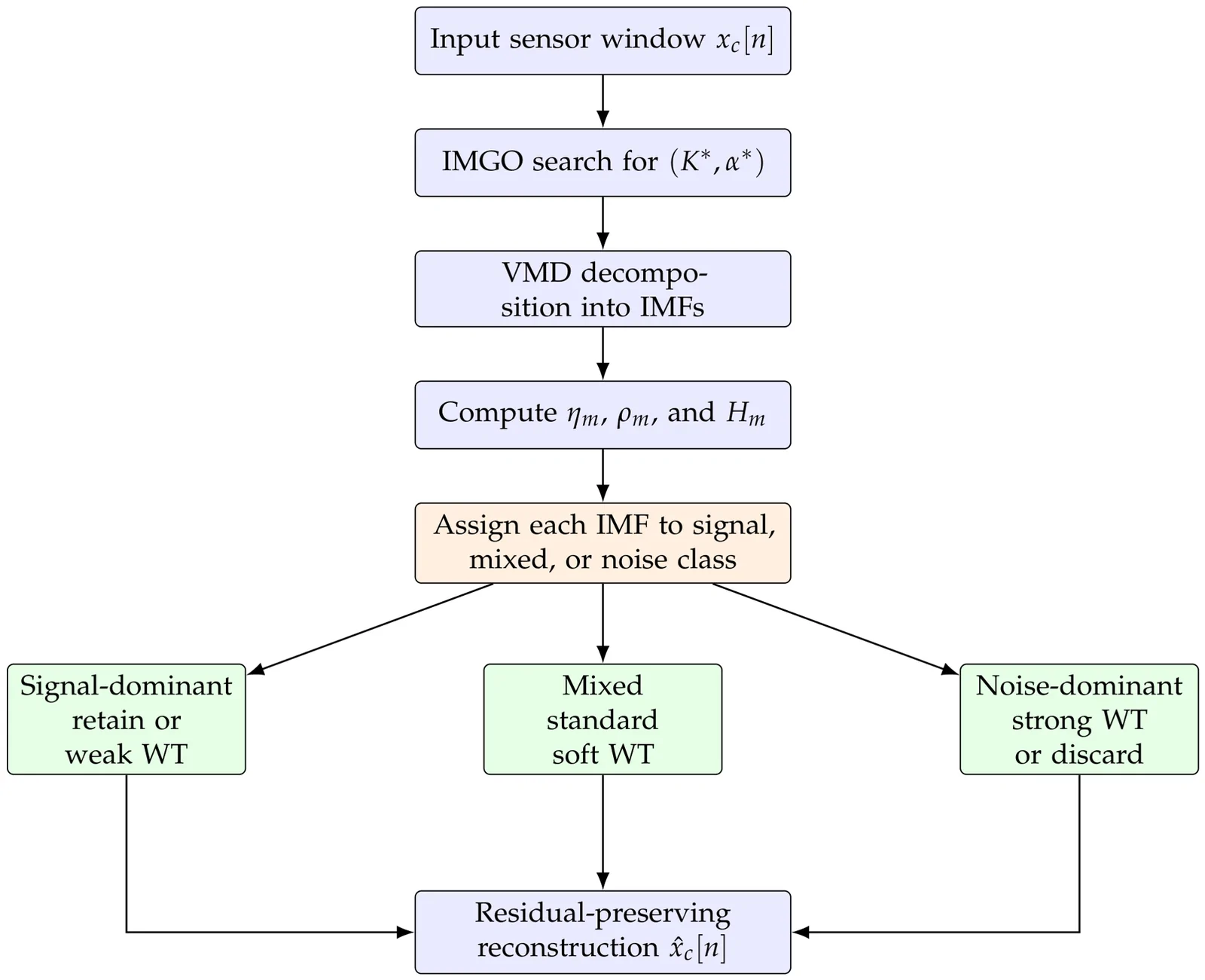
Mode-level differentiated processing in the IMGO–VMD–WT module.

The coefficient β gives the denoised reconstruction of the larger weight while retaining the original trend component, which is important for preserving material-sensitive time-domain statistics. The value β=0.59 was also adopted through repeated trials as a recommended value for laboratory experiments; when formally applied in engineering, it can be adjusted and determined according to site conditions. Reconstruction behavior and downstream time-domain feature stability were considered. The screening preceded outer five-fold evaluation, and no outer test window was used; β was then fixed for all methods and folds. Figure 2 illustrates the mode-level grading and reconstruction procedure used after VMD parameter selection.

### 2.6. Feature Extraction and Classification

For each processed window and each channel, eight time-domain features were extracted: mean, standard deviation, root mean square, minimum, maximum, median, skewness, and kurtosis. These features are concatenated across the eleven channels, giving 88 features per window. A data-quality check showed no non-finite values and no zero-variance channel windows among the 245 windows. Consequently, zero-value replacement was not required in the present dataset.

Three classifiers are evaluated: radial-basis-function support vector machine (RBF-SVM), nearest centroid, and random forest [24,25,26]. RBF-SVM is used as the primary recognition classifier because it can model nonlinear boundaries in the compact 88-dimensional feature space. The nearest-centroid classifier is reported as a conservative feature-space probe, and random forest is reported as a high-capacity comparison. The implementation usesNumPy, SciPy, PyWavelets, and scikit-learn [27,28,29,30].

## 3. Experimental Platform, Dataset, and Evaluation Protocol

The experiments used test blocks prepared according to representative coal and rock properties, either independently or in combination, to simulate typical material combinations encountered during drilling. This laboratory configuration reproduces key drilling-response characteristics, although it cannot fully represent the uneven and discontinuous geological structure of field coal seams. The experiment provides a controlled data basis for evaluating coal–rock identification from drilling sensor signals. Table 1 separates measurement, processing, response, and evaluation so that the reproducibility boundary is explicit.

### 3.1. Self-Built Simulation Drilling Platform

**Figure 3:**
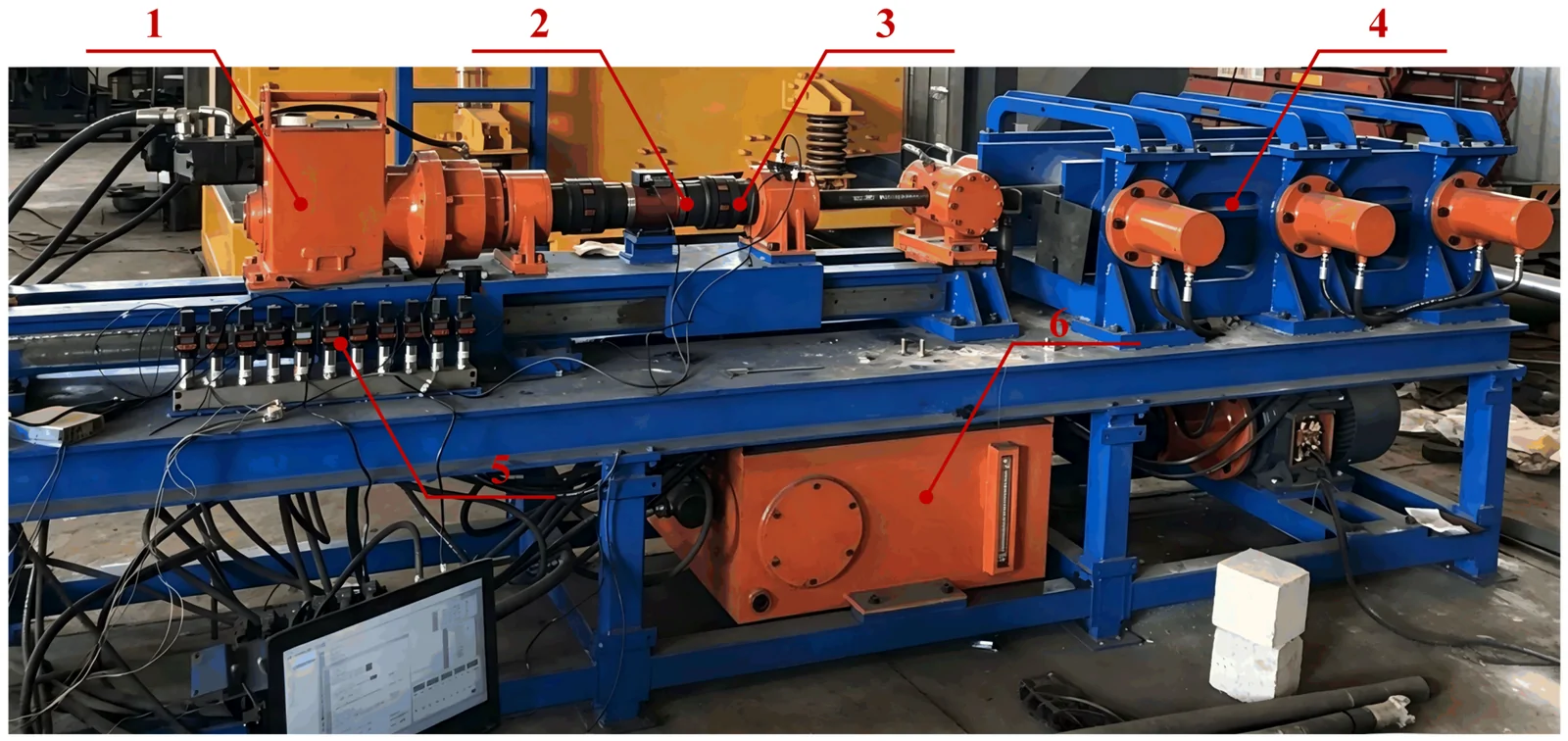
Schematic of the self-built simulation drilling platform used in the experiments. The numbered structures are as follows: 1, drilling drive and feed module; 2, drill rod and spindle axis; 3, coal–rock specimen fixture; 4, loading cylinder and end cap; 5, sensor manifold; 6, hydraulic power unit.

The drilling data were acquired using the self-built simulation drilling platform built by the research group and installed at the Wenchang Campus of China University of Mining and Technology. The platform simulates the drilling-tool section of a mine drilling machine during borehole formation. Figure 3 shows the platform and its main functional modules. The synchronized export contains eleven channels at 1000 samples/s. The drill string rotational speed was set to 110 r/min, and the feed rate was set to 1 m/min. The length of the drill string was 1 m, and the drill bit was a double-helix type with a drilling diameter of 50 mm. The acquisition included one run each for C1, C2, C3, and C6; three runs for C4; and two runs for C5. These records were used to analyze drilling status and coal–rock identification performance. The rated propulsion pressure was 13 MPa and the rated rotation pressure was 16 MPa. The prepared test blocks had dimensions of 350 mm × 350 mm × 750 mm.

### 3.2. Sensor Channels and Dataset Organization

The primary classification corpus contains 247,680 sensor sampling points and eleven channels: pump outlet pressure, multi-way-valve inlet pressure, propulsion-cylinder inlet pressure, propulsion-cylinder outlet pressure, motor inlet pressure, motor outlet pressure, loading-cylinder inlet pressure, loading-cylinder outlet pressure, laser displacement, torque, and rotational speed. The six labels are C1 sandstone, C2 shale, C3 mudstone, C4 bituminous coal, C5 anthracite, and C6 lignite. At 1000 samples/s, non-overlapping one-second windowing retained 245 complete windows. Residual samples shorter than one second at the ends of the selected records were not used for classification. Table 2, Table 3, Table 4 and Table 5 summarize the data boundary.

**Figure 4:**
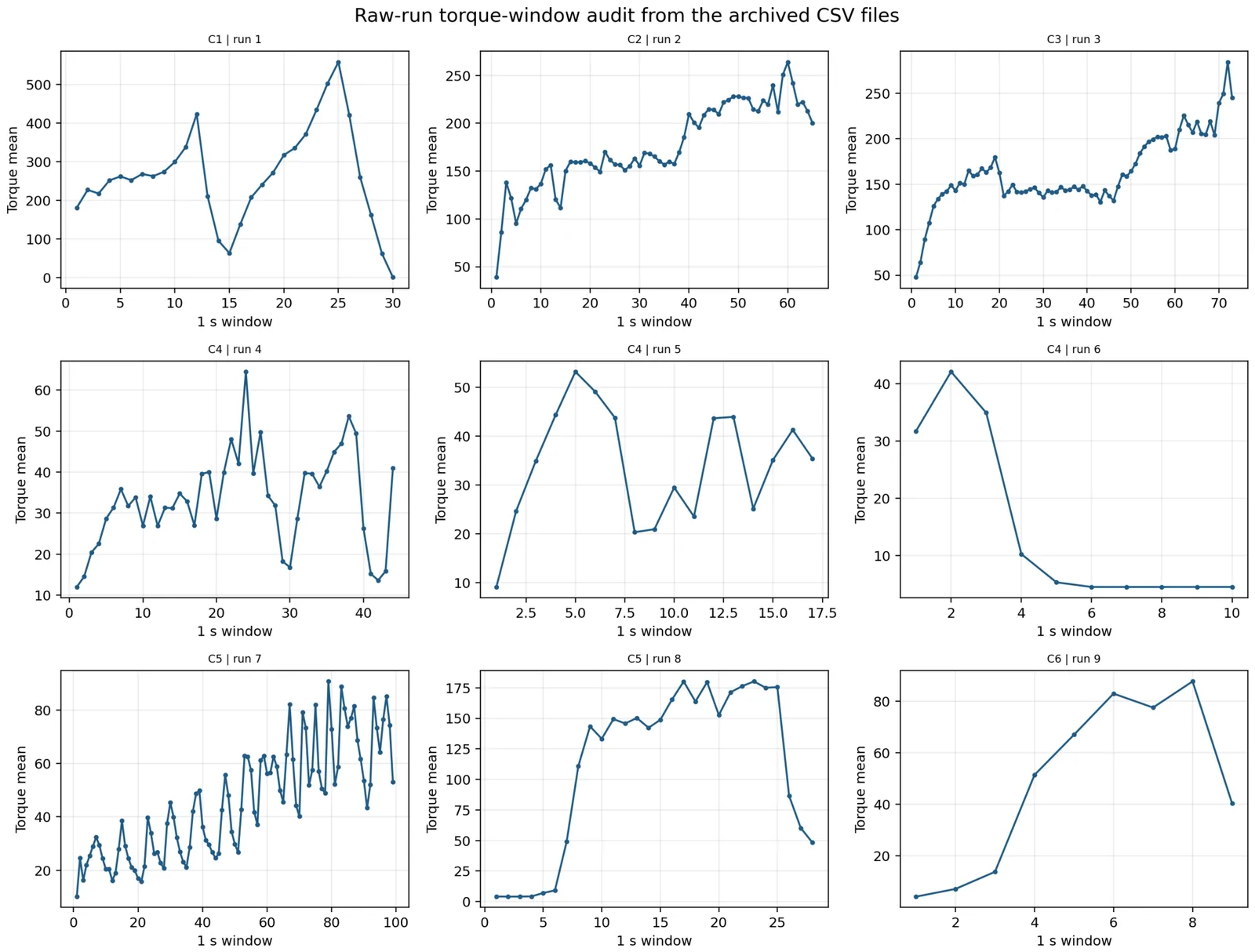
Torque means for the labeled raw drilling runs.

Figure 4 visualizes the torque-window means of the labeled raw runs and shows the operating differences among available records.

**Figure 5:**
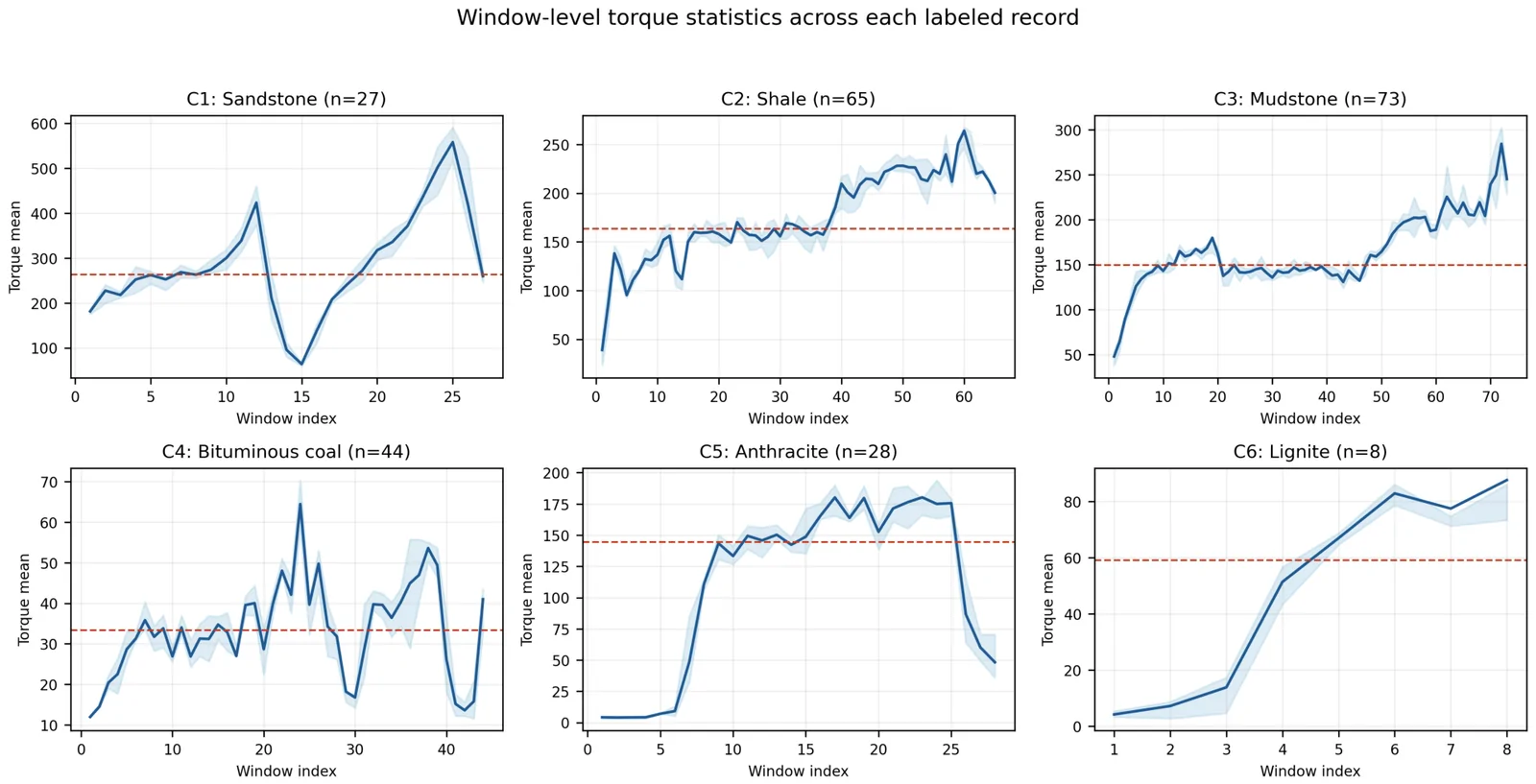
Window-level torque mean and interquartile range for each labeled record.

Figure 5 summarizes the window-level torque variation in the selected labeled records.

### 3.3. Evaluation Protocol

The evaluation was designed to compare whether the proposed adaptive signal-processing module improves downstream coal–rock recognition while retaining a measurable denoising effect. Six input methods were therefore evaluated under the same window definition, feature set, classifier settings, and data partitioning strategy:

1. Raw-feature baseline: The features were extracted directly from the original eleven-channel windows without denoising or decomposition.
2. Wavelet-thresholding baseline: Each channel was processed by conventional wavelet-threshold denoising before feature extraction, providing a single-stage denoising reference.
3. Fixed VMD–WT baseline: VMD–WT was performed with fixed parameters K=4 and α=2000, so that the effect of decomposition-based denoising could be separated from the effect of adaptive parameter optimization.
4. PSO–VMD–WT: PSO selected the window-level VMD parameters under the same objective, search range, and evaluation budget as the proposed adaptive comparison.
5. GWO–VMD–WT: GWO selected the window-level VMD parameters under the same objective, search range, and evaluation budget as the proposed adaptive comparison.
6. Proposed IMGO–VMD–WT: IMGO selected window-level VMD parameters using the torque channel, after which IMF grading, differentiated wavelet-threshold processing, residual-preserving reconstruction, and eleven-channel feature extraction were performed.

The optimizer-comparison experiments used a StandardScaler–RBF-SVC implementation with C=10 and γ=scale. For comparative evaluation of optimizer selection, two conventional population-based baselines, PSO and GWO, were implemented for the same two-dimensional VMD search problem. Both baselines used the torque channel, the fitness function in Equation (4), the search space K∈[3,6] and α∈[400,4000], a population of eight, and eight search rounds.

For PSO, the particle position $z_{i}$ and velocity $v_{i}$ were updated by

(15) $$v_{i}^{\left(r+1\right)}=0.72v_{i}^{\left(r\right)}+1.49q_{1,i}^{\left(r\right)}⊙\left(p_{i}-z_{i}^{\left(r\right)}\right)+1.49q_{2,i}^{\left(r\right)}⊙\left(g-z_{i}^{\left(r\right)}\right),\;z_{i}^{\left(r+1\right)}=\Pi_{\Omega }\left(z_{i}^{\left(r\right)}+v_{i}^{\left(r+1\right)}\right),$$

where $p_{i}$ is the particle’s best position, g is the population’s best position, $q_{1,i}$ and $q_{2,i}$ are independent uniform random vectors, and $\Pi_{\Omega }$ clips the position to the feasible search space. The velocity limit was set to 25% of the corresponding search-range width.

For GWO, the three best wolves were denoted by $z_{\alpha }$, $z_{\beta }$, and $z_{\delta }$. At round *r*, each wolf was updated using

(16) $$z_{i}^{\left(r+1\right)}=\frac{1}{3}\sum_{\ell \in \{\alpha ,\beta ,\delta \}}\left[z_{\ell }^{\left(r\right)}-A_{\ell }^{\left(r\right)}⊙\left|C_{\ell }^{\left(r\right)}⊙z_{\ell }^{\left(r\right)}-z_{i}^{\left(r\right)}\right|\right],\;A_{\ell }^{\left(r\right)}=2a^{\left(r\right)}q_{1,\ell }^{\left(r\right)}-a^{\left(r\right)},\;C_{\ell }^{\left(r\right)}=2q_{2,\ell }^{\left(r\right)},$$

where $a^{\left(r\right)}$ decreases linearly from 2 to 0 over the eight rounds. After each position update, *K* is rounded to the nearest feasible integer and α is clipped to its search interval. The initial population was generated with a fixed seed for reproducibility. Each algorithm used eight population evaluations in each of eight rounds, giving 64 fitness evaluations per window.

The classification experiment used five-fold contiguous-block validation rather than random splitting. For each material category, the one-second windows were kept in their original temporal order and divided into five non-overlapping contiguous blocks. In each fold, one block from every category was used only for testing and the remaining four blocks were used for training. All input methods used the same fold assignments. Feature standardization, where used by the classifier, was fitted on the training windows of each fold and then applied to that fold’s test windows. The fixed VMD–WT parameters, PSO/GWO search ranges and budgets, IMGO search range, fitness weights, IMF-score weights, thresholds, and β were fixed before the fold evaluation, and they were not re-optimized using test windows. The final classification results are reported as the mean and standard deviation across the five folds.

Overall accuracy, macro-F1, and the confusion matrix were used as the primary recognition metrics. Accuracy measures the proportion of correctly classified windows:

(17) $$\mathrm{Accuracy}=\frac{\sum_{c=1}^{C}TP_{c}}{N},$$

where C=6 is the number of material categories, $TP_{c}$ is the number of correctly classified windows in category *c*, and *N* is the total number of test windows. Because the dataset is imbalanced, macro-F1 was used to give equal weight to each material category. For category *c*, precision, recall, and F1-score are defined as

(18) $$P_{c}=\frac{TP_{c}}{TP_{c}+FP_{c}},\;R_{c}=\frac{TP_{c}}{TP_{c}+FN_{c}},\;F1_{c}=\frac{2P_{c}R_{c}}{P_{c}+R_{c}},$$

and macro-F1 is calculated as

(19) $$\mathrm{Macro}-F1=\frac{1}{C}\sum_{c=1}^{C}F1_{c}.$$

The confusion matrix was further used to identify which material categories were most frequently confused and to determine whether preprocessing changed the distribution of classification errors rather than only the aggregate score.

For real drilling measurements, a clean noise-free reference signal is unavailable. Therefore, signal-to-noise ratio (SNR) and root mean square error (RMSE) were not calculated on the unaltered measurements. Instead, a controlled denoising test was conducted by selecting twelve measured torque windows, two from each material category, and adding Gaussian noise scaled to an input SNR of 10 dB. The original measured window x[n] was treated as the reference, the noisy input was processed by each denoising method, and the reconstructed output was denoted as $\hat{x}\left[n\right]$. The output SNR, SNR gain, and RMSE were calculated as

(20) $${\mathrm{SNR}}_{\mathrm{out}}=10\log_{10}\left(\frac{\sum_{n}x^{2}\left[n\right]}{\sum_{n}{\left(x\left[n\right]-\hat{x}\left[n\right]\right)}^{2}}\right),\;\Delta \mathrm{SNR}={\mathrm{SNR}}_{\mathrm{out}}-10,$$

(21) $$\mathrm{RMSE}=\sqrt{\frac{1}{L}\sum_{n=1}^{L}{\left(x\left[n\right]-\hat{x}\left[n\right]\right)}^{2}},$$

where *L* is the window length. These metrics quantify the reconstruction quality in the controlled test, whereas the classification metrics quantify whether the processed signals preserve material-discriminative information.

## 4. Results

In this section, a statistical analysis of the previous experimental data is conducted to objectively evaluate the algorithm proposed in this paper. However, it is important to note that we conducted the experiments and analysis under limited data conditions. Moreover, the limitations of this article are also discussed in this section.

### 4.1. Classification Performance of Processed Multi-Source Features

The complete experiment used 245 one-second windows and 88 features per window. All input conditions were processed using the same retained windows, feature extraction procedure, classifier settings, contiguous-block fold assignments, and optimizer evaluation budget. Table 6 reports the primary RBF-SVM results under the five-fold contiguous block validation protocol. An exploratory paired fold-level comparison between fixed VMD–WT and IMGO–VMD–WT was performed using the same five contiguous folds. The analysis gives an accuracy difference of 0.40 percentage points and a macro-F1 difference of 0.49 percentage points. The corresponding exact sign-permutation *p*-values are 0.6875 and 0.5625, with bootstrap 95% intervals of −0.73 to 1.73 percentage points and −0.50 to 1.65 percentage points, respectively. The raw-feature baseline reached 90.26% accuracy and 78.34% macro-F1, showing that the eleven-channel signals themselves are informative. WT and fixed VMD–WT increased the aggregate RBF-SVM scores to 91.94%/80.37% and 92.17%/81.73%, respectively. PSO–VMD–WT and GWO–VMD–WT both reached 92.13% accuracy and 82.17% macro-F1. Tracking of the optimization iterations showed that because both algorithms optimized the same VMD objective in the same low-dimensional search space, they converged prematurely to the same local optimum in different generations. This premature convergence produced identical selected VMD parameters and consequently identical fold-level classification summaries after feature extraction. By contrast, IMGO uses diversified initialization, representative anchor candidates, elite-guided movement, adaptive diffusion and reproduction, and stagnation perturbation to maintain population diversity during the search. These mechanisms reduced the observed tendency toward severe premature convergence in the present parameter-selection task. The proposed IMGO–VMD–WT method reached 92.57% accuracy and 82.22% macro-F1, giving the highest mean score among the six listed inputs.

**Figure 6:**
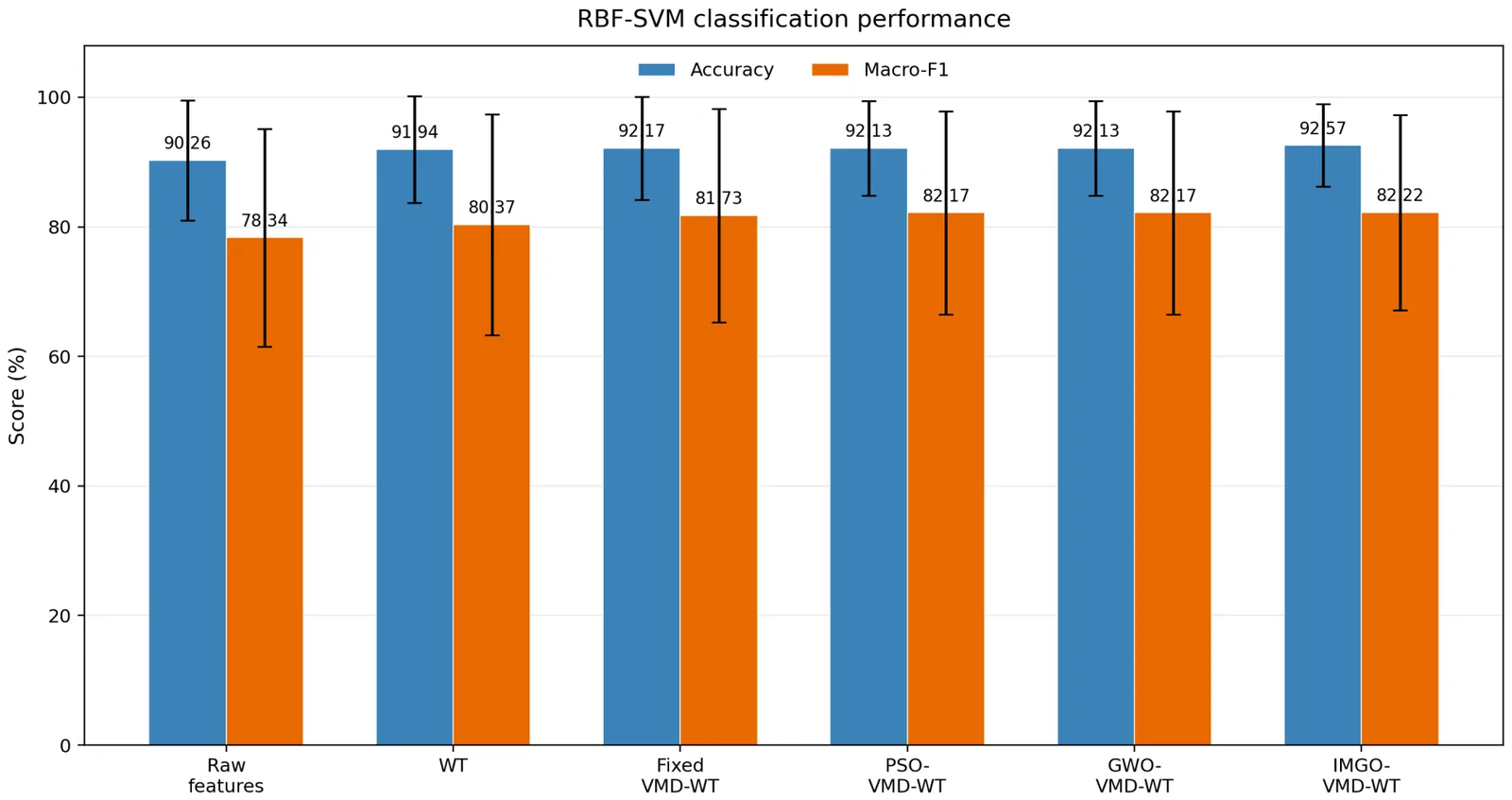
RBF-SVM classification performance of raw and processed eleven-channel features.

Figure 6 provides the corresponding visual comparison of the primary RBF-SVM results.

### 4.2. Optimizer Comparison

To compare optimizer selection on the application problem, PSO–VMD–WT and GWO–VMD–WT were evaluated on all 245 complete one-second windows using the same torque-channel objective, parameter space, population size, eight search rounds, IMF grading, wavelet configuration, residual-preserving reconstruction, and contiguous-block fold assignments. Each optimizer therefore used 64 objective evaluations per window. The resulting features were classified with StandardScaler–RBF-SVC (C=10; γ=scale).

Table 7 reports the two secondary classifiers. The nearest-centroid model serves as a conservative feature-space probe, while random forest checks whether the same preprocessing gains remain visible under a higher-capacity learner. The PSO–VMD–WT and GWO–VMD–WT rows use features generated under the same objective and search budget as the primary comparison. Relative to the raw-feature baseline, the processed inputs improve nearest-centroid accuracy and macro-F1, while random forest nearly saturates on the current dataset. These results suggest that the multi-source features contain strong class information once a flexible classifier is available, but they also reinforce the risk of overfitting. They support the use of RBF-SVM as the primary classifier because it can reveal preprocessing effects without saturating on the current dataset.

To examine whether the reported performance depends mainly on the original eight time-domain descriptors, an auxiliary 100-feature analysis was performed. The results is shown in Table 8. The additional feature set included six pressure-difference features, two torque-to-speed ratio features, two displacement-rate features, and six spectral descriptors derived from the torque and speed channels. Under the same contiguous-block protocol, an RBF-SVM reached 95.82±5.51% accuracy and 89.98±12.98% macro-F1, whereas a random forest reached 100.00% for both metrics in all five folds. These results are reported as an auxiliary analysis rather than as a replacement for the primary experiment, because the perfect random-forest score and the strong correlation structure among the 100 descriptors indicate a material risk of overfitting on this single-run-per-class corpus.

The torque-only analysis provides a deliberately conservative baseline for the role of the proposed multi-source feature set. Using the same five contiguous blocks and a nearest-centroid classifier, torque-only features reached 51.37±12.31% accuracy and 43.95±11.04% macro-F1; the corresponding random-forest values were 58.35±12.87% and 50.05±17.98%. The gap between this analysis and the main multi-source results supports the interpretation that the classification signal is distributed across the sensor channels, while it also reinforces the need for independent-run and independent-specimen validation.

**Figure 7:**
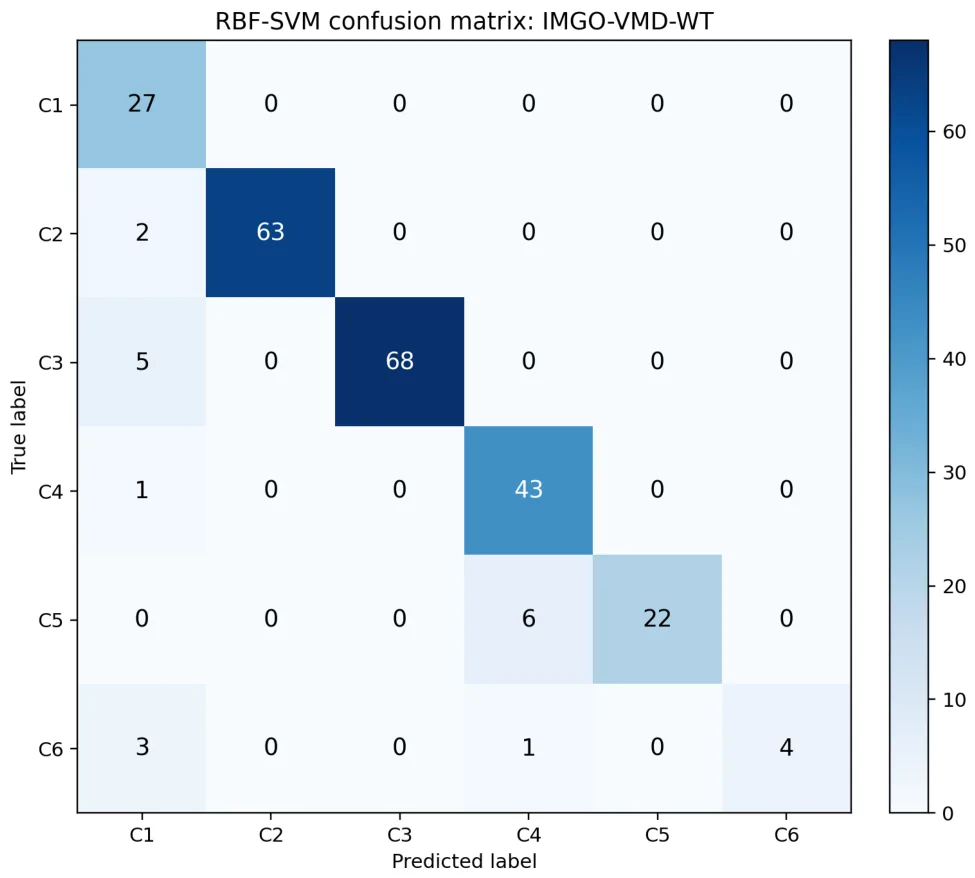
Confusion matrix of IMGO–VMD–WT features with the RBF-SVM classifier. Rows are true labels and columns are predicted labels.

The confusion matrix for IMGO–VMD–WT with the RBF-SVM classifier is shown in Figure 7 and the evaluation indexes is shown in Table 9. C1–C4 have a high recall, whereas C5 and especially C6 are less stable, with recalls of 78.6% and 50.0%, respectively. The main off-diagonal errors occur in C2/C1, C3/C1, C5/C4, and C6/C1. The aggregate confusion matrix contains 227 correct predictions out of 245 windows, corresponding to 92.65% aggregate accuracy; this value differs slightly from the fold-averaged accuracy in Table 6 because the table reports the mean across folds. Because C6 has only eight windows, the C6 recall and F1-score are sensitive to a small number of predictions.

### 4.3. Signal Processing Behavior of the Proposed Module

**Figure 8:**
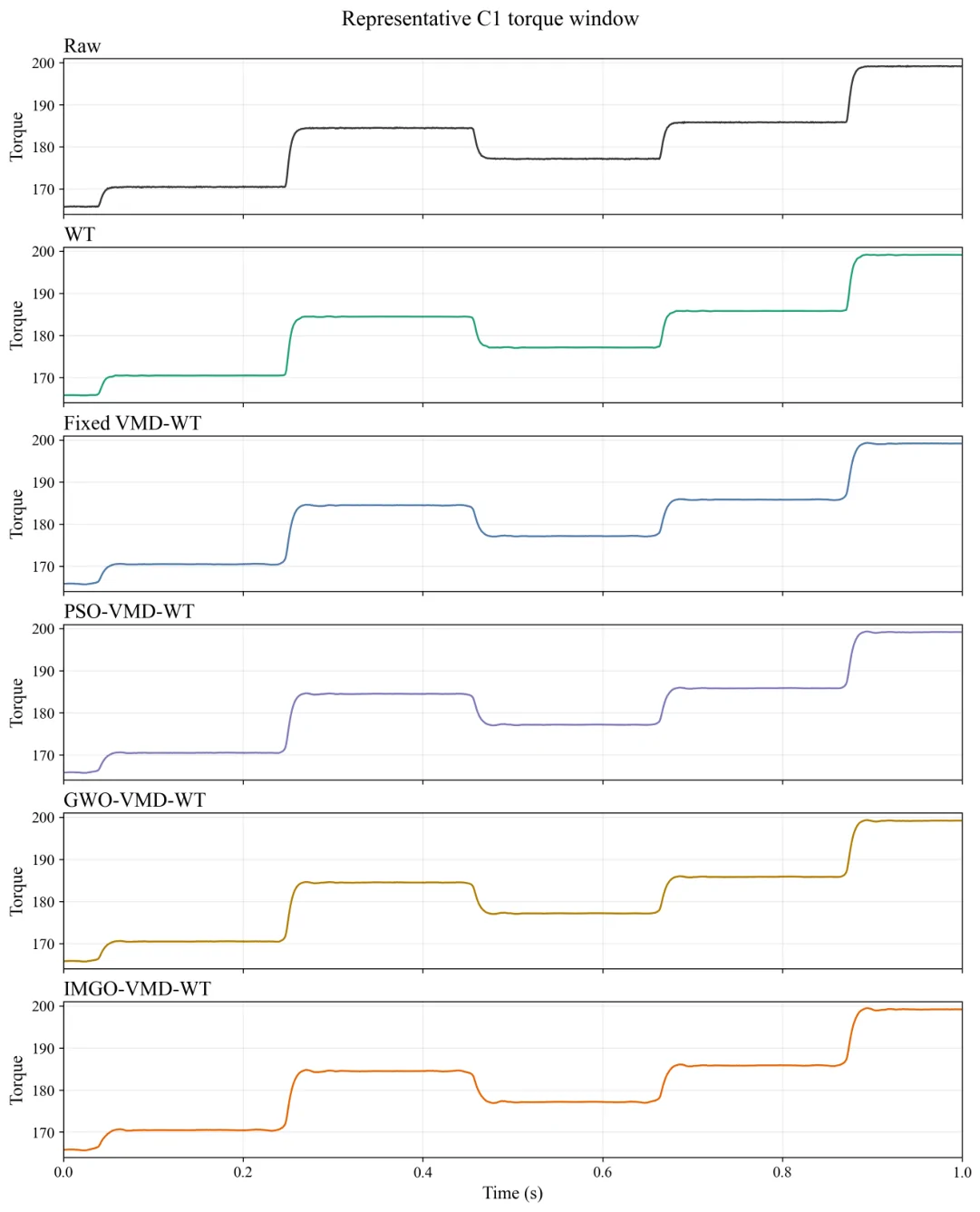
Representative torque signal processing result for a C1 window. From top to bottom, the panels show the raw signal, WT output, fixed VMD–WT output, PSO–VMD–WT output, GWO–VMD–WT output, and IMGO–VMD–WT output.

**Figure 9:**
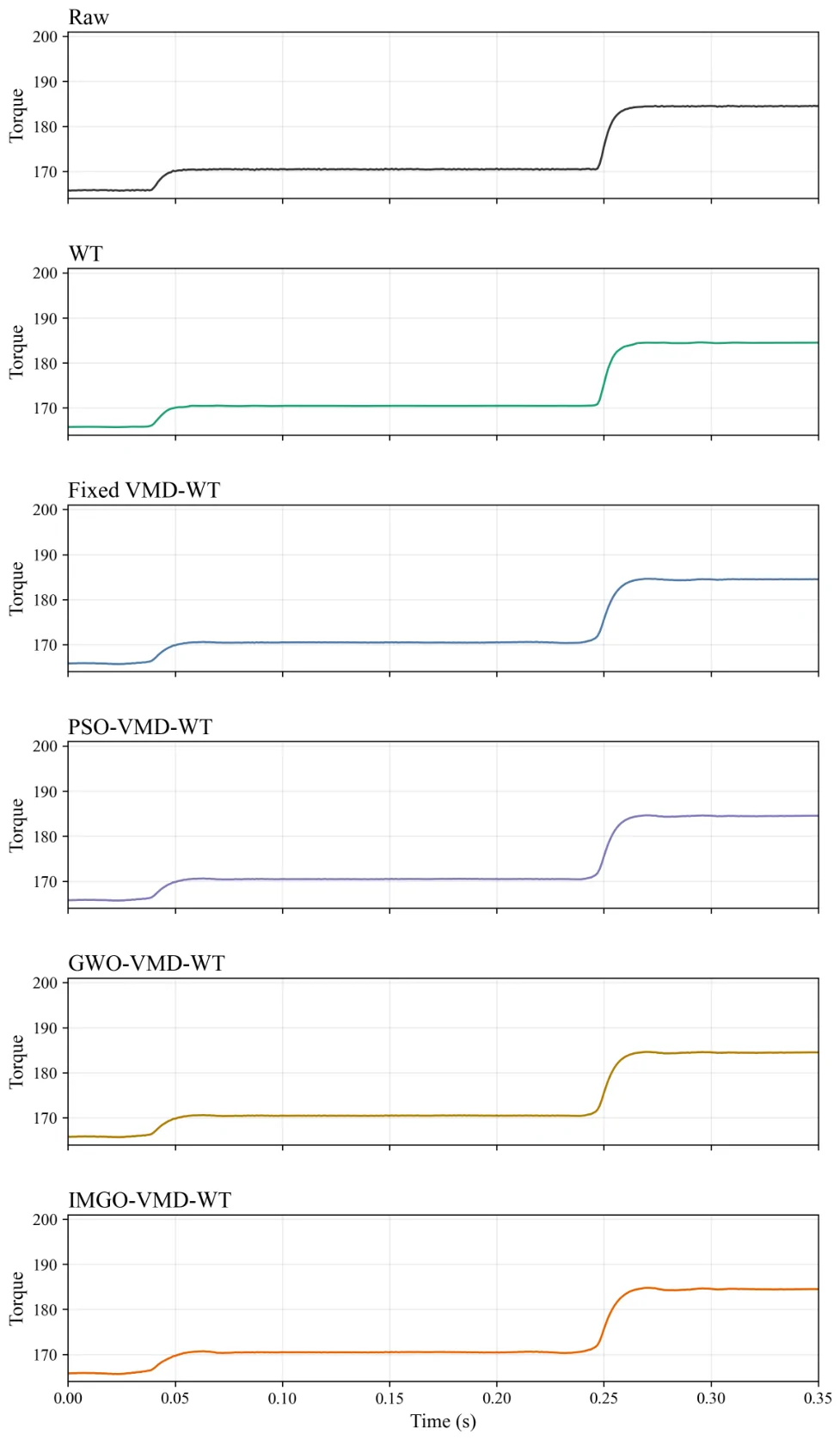
Detailed view of the representative torque window around the major load-transition region, illustrating edge preservation and residual reconstruction.

Figure 8 shows a representative C1 torque window. Both WT and VMD-based methods reduce high-frequency fluctuations while preserving the major load transitions. The panels are ordered as raw, WT, fixed VMD–WT, PSO–VMD–WT, GWO–VMD–WT, and IMGO–VMD–WT. The PSO–VMD–WT and GWO–VMD–WT panels use the corresponding optimizer-selected parameters for the same window and exhibit the same major load transitions. For this window, IMGO selected K=5 and α=3581. The proposed output remains close to the measured step-like torque trend because residual-preserving reconstruction retains 41% of the original signal while using 59% of the VMD–WT reconstruction. In other words, the method smooths the fine-scale fluctuations without erasing the coarse load steps that encode the drilling state. Figure 9 gives an enlarged view of the major load-transition region.

**Figure 10:**
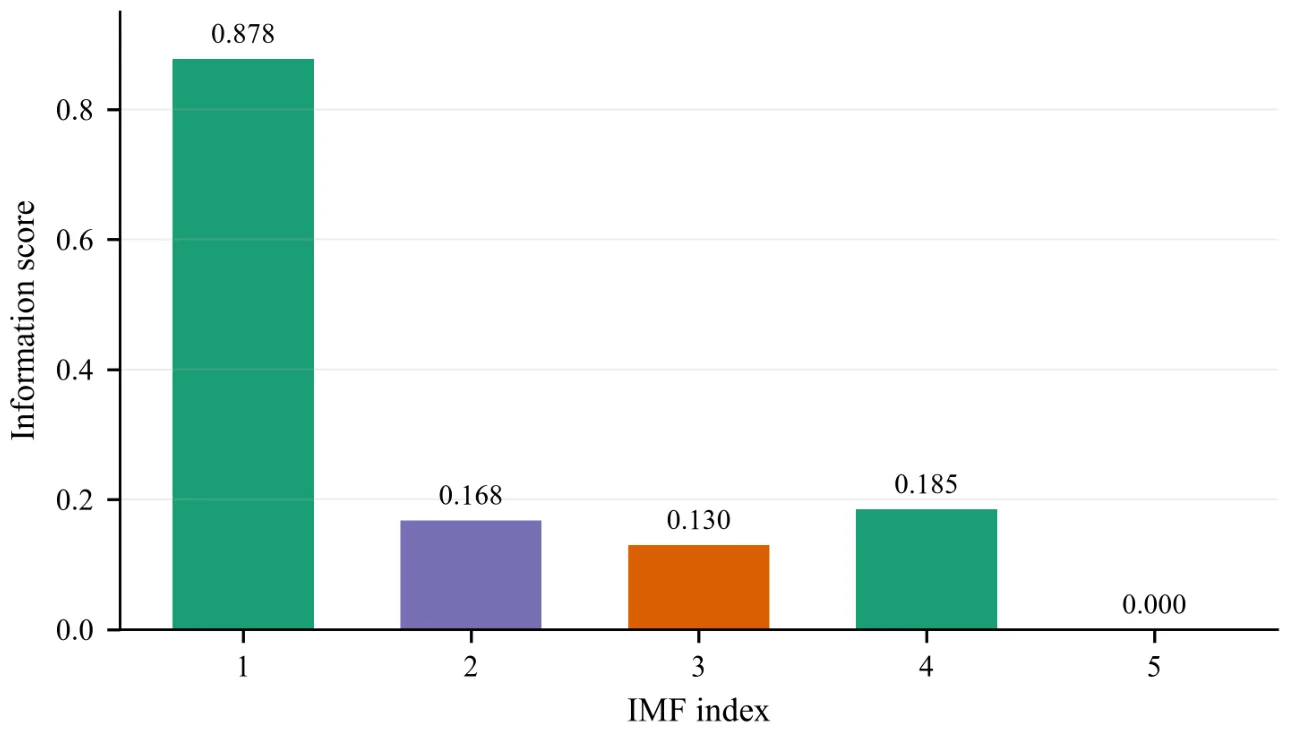
IMF information scores for the representative C1 torque window processed by IMGO–VMD–WT. Higher scores indicate greater energy contribution, stronger correlation with the original signal, and lower envelope complexity.

The corresponding IMF information scores are shown in Figure 10. The first IMF is the dominant signal-bearing component, while the remaining modes have lower fused scores. This confirms that the mode scoring module separates high-information and low-information components, allowing the reconstruction to preserve material-sensitive load variation while suppressing weaker fluctuation components.

### 4.4. Controlled Noise-Injection Denoising Test

Because the real drilling measurements do not have clean reference signals, SNR and RMSE are not computed directly on the unaltered measurements. Instead, twelve measured torque windows, two from each material, were used as references and contaminated with 10 dB additive Gaussian noise. Table 10 summarizes the denoising results. WT and fixed VMD–WT increased the mean output SNR to 17.95 dB and 19.11 dB, respectively. PSO–VMD–WT and GWO–VMD–WT produced similar denoising results under the same test setting. The proposed IMGO–VMD–WT method produced the lowest mean RMSE among the tested methods, at 1.379, and reached 19.19 dB mean output SNR. Notably, this method improves SNR while preserving recognition-relevant signal structure under a common comparison protocol.

**Figure 11:**
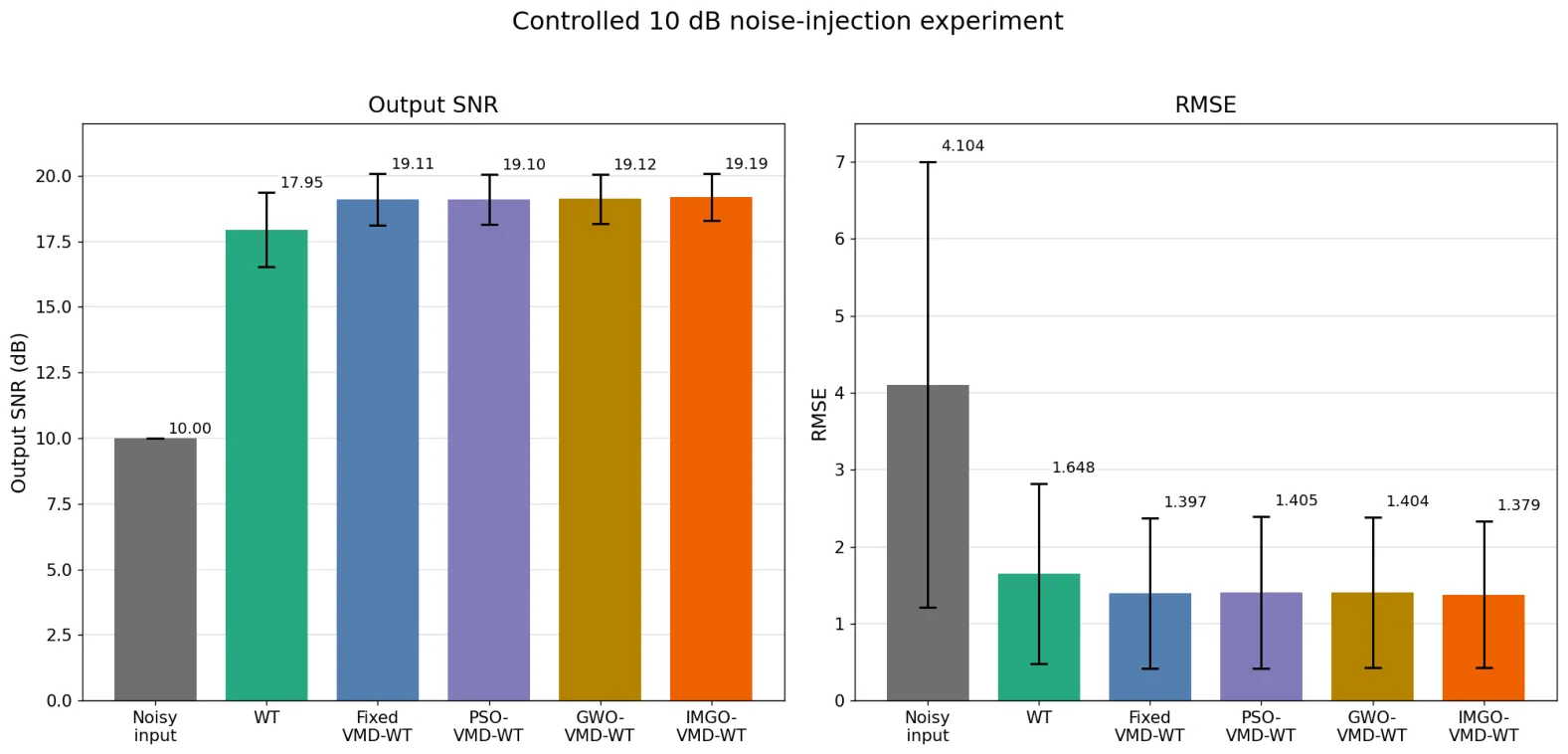
Output SNR and RMSE in the controlled 10 dB noise-injection experiment. Error bars show one standard deviation across 12 measured torque windows.

Figure 11 plots the same noise-injection results with one-standard-deviation error bars.

## 5. Discussion

The experimental results indicate that the eleven-channel drilling signals contain material-discriminative information even before signal reconstruction. The raw-feature RBF-SVM baseline reached 90.26% accuracy and 78.34% macro-F1, showing that the pressure, displacement, torque, and speed channels jointly describe the drilling response of different coal–rock materials. After preprocessing, WT, fixed VMD–WT, PSO–VMD–WT, GWO–VMD–WT, and IMGO–VMD–WT all improved the primary classification metrics to different degrees. The proposed IMGO–VMD–WT method obtained the highest mean accuracy and macro-F1 among the six input methods, but the gain over fixed VMD–WT and the two population-optimization baselines was modest. This also reveals the limitations of this study. That is to say, we need to acknowledge that due to the constraints of the experimental conditions, the experimental results lead to the following conclusion: the exploratory paired fold-level comparison between fixed VMD–WT and IMGO–VMD–WT did not show a statistically significant difference under the five contiguous folds. Therefore, the main implication of the classification experiment is that decomposition-aware denoising and residual-preserving reconstruction can stabilize multi-source feature extraction under limited data, whereas superiority among closely related optimized VMD–WT variants requires larger grouped datasets for confirmation.

The optimizer comparison further clarifies the role of adaptive VMD parameter selection. PSO, GWO, and IMGO were evaluated under the same VMD parameter space, population size, search budget, objective function, IMF grading rule, wavelet configuration, and fold assignment. The three optimized VMD–WT variants produced similar RBF-SVM scores, indicating that for the current two-parameter search problem, most of the performance gain comes from introducing adaptive decomposition and mode-wise processing rather than from a large separation among population-search algorithms. The identical PSO and GWO classification summaries are consistent with premature convergence, a known limitation of population-based swarm optimizers. The iteration traces showed that both algorithms, while optimizing the same objective, became trapped in the same local optimum at different generations and therefore produced the same selected VMD parameters for downstream reconstruction and classification. In contrast, the IMGO design combines broad initial coverage, anchor-based candidate preservation, elite-guided local movement, adaptive diffusion and reproduction, and perturbation after stagnation. This balance between exploration and exploitation helps preserve search diversity and reduces the likelihood of severe premature convergence. The higher RBF-SVM accuracy and lower controlled-noise RMSE obtained by IMGO–VMD–WT are consistent with this search behavior, although larger grouped datasets are still required to establish a general optimizer-level advantage.

The secondary classifier and feature-analysis results should be interpreted together with the limited-data setting. The nearest-centroid classifier produced lower scores than RBF-SVM and random forest, which is expected because it imposes a simple class-mean structure on the feature space. Random forest reached very high or perfect performance in several settings, especially when the auxiliary 100-feature set was used. Such results confirm that the measured channels carry strong class information, but they also indicate a risk of fitting record-specific patterns in a dataset with only 245 complete windows and uneven class support. The torque-only analysis yielded substantially lower scores than the multi-source feature set, supporting the use of synchronized pressure, displacement, torque, and speed responses rather than relying on a single load channel.

The signal-processing figures and controlled denoising test explain how the proposed module affects the measured drilling responses. The representative torque window shows that VMD–WT-based methods suppress fine-scale fluctuations while retaining the main step-like load transitions. The IMF information scores concentrate the dominant signal contribution in the high-scoring modes, which supports the use of energy, correlation, and envelope-entropy indicators for differentiated mode treatment. In the 10 dB noise-injection experiment, IMGO–VMD–WT produced a mean output SNR of 19.19 dB and a mean RMSE of 1.379, slightly outperforming the tested alternatives. Because the reference in this test is the measured torque signal before synthetic noise injection, these metrics quantify recovery from controlled Gaussian perturbation. They do not fully represent performance under all hydraulic, mechanical, and geological disturbances encountered in field drilling.

It must be emphasized that although the method proposed in this paper has been proven to have certain potential advantages through experiments, further experiments are still needed to provide a more comprehensive proof. Several limitations define the scope of the present conclusions. First, the primary corpus contains 245 complete one-second windows from 247,680 labeled samples, with only eight windows in class C6. The macro-F1 score and the C6 entries in the confusion matrix are therefore sensitive to a small number of predictions. Second, the contiguous-block protocol reduces random adjacency leakage but remains a within-record validation design. The raw-run statistics show that repeated runs are incomplete across classes and that repeated C4 and C5 records can differ markedly in their operating characteristics. Consequently, the current results should not be interpreted as independent-specimen or cross-site generalization. Third, the reported timing shows that the present implementation is more suitable for offline analysis than for direct real-time control. Fourth, the noise-injection experiment uses synthetic Gaussian noise on measured torque windows and does not include independently marked coal–rock interfaces or clean field references. Future work should extend the dataset by collecting multiple specimens and independent drilling runs for each material under controlled variations in bit condition, feed rate, axial load, rotation speed, and coal–rock interface structure. Validation should use run-level or specimen-level grouped splits, paired statistical comparisons, and pre-specified metrics. For engineering deployment, the adaptive decomposition stage should be accelerated or approximated for target hardware, and the processed signals should be evaluated against independently marked interfaces and field labels. These steps are necessary to determine whether the laboratory-scale gains observed here can be maintained under realistic geological variability.

## 6. Conclusions

This study developed an adaptive multi-source drilling-signal processing framework for coal–rock identification under limited laboratory data. The proposed IMGO–VMD–WT chain uses torque-guided VMD parameter selection, IMF information scoring, differentiated wavelet-threshold processing, residual-preserving reconstruction, and eleven-channel feature extraction before classification. Under five-fold contiguous-block validation on 245 complete one-second windows from six material categories, IMGO–VMD–WT achieved 92.57% accuracy and 82.22% macro-F1 with the primary RBF-SVM classifier. Compared with raw features, the accuracy increased by 2.31 percentage points and macro-F1 increased by 3.88 percentage points. PSO–VMD–WT and GWO–VMD–WT achieved 92.13% accuracy and 82.17% macro-F1 under the same stated search budget, indicating that optimized VMD–WT preprocessing provides a consistent improvement over raw and single-stage WT inputs in the present dataset.

The controlled 10 dB noise-injection experiment further showed that the proposed reconstruction strategy can suppress added noise while preserving major torque-load transitions. Across twelve measured torque windows, IMGO–VMD–WT obtained a mean output SNR of 19.19 dB, a mean SNR gain of 9.19 dB, and a mean RMSE of 1.379. Together with the signal examples and IMF-score analysis, these results support the feasibility of using decomposition-aware, mode-differentiated processing for limited-data drilling sensor analysis.

The conclusions are limited to the available laboratory corpus. The dataset remains small and imbalanced, the primary validation is within-record rather than grouped by independent specimen or drilling run, and the present computational cost does not yet meet real-time control requirements. Future studies should collect larger multi-run datasets, evaluate grouped generalization across specimens and operating conditions, reduce the runtime of adaptive decomposition, and validate the reconstructed signals against independently marked coal–rock interfaces before field deployment.

## Figures and Tables

**Table 1:** DOE-style description of the measurement, processing, response, and evaluation units.

Item	Definition	Recorded Setting	Reproducibility Boundary
Objective	Identify one of six material labels from synchronized drilling signals	C1 sandstone, C2 shale, C3 mudstone, C4 bituminous coal, C5 anthracite, C6 lignite	Validated only on the supplied laboratory platform and labeled records
Input	Eleven channels, 1000 samples/s, non-overlapping 1 s windows	1000 samples per window; pressure, displacement, torque, and speed channels	/
Response	Window-level material label and derived signal-quality metrics	Accuracy and macro-F1 in [0,1]; SNR in dB; RMSE in the torque channel’s acquisition units	/
Variability treatment	Contiguous-block folds, same folds for all methods, controlled 10 dB Gaussian-noise test	Five blocks per class; classifier scaling fit within each training fold	/

**Table 2:** Labeled raw drilling runs in the acquisition folder. Rows/windows are reported for each file; the primary classification corpus uses one selected complete-window segment per class, as summarized in Table 3.

Class	Labeled Runs	Rows/Windows per Run	Torque-Window Median	Speed-Window Median
C1	1	30,000/30	260.86	110.74
C2	1	65,800/65	163.24	109.90
C3	1	73,400/73	149.50	110.36
C4	3	44,600/44; 17,200/17; 10,400/10	33.35; 35.13; 4.91	109.05; 109.51; 17.19
C5	2	99,000/99; 28,000/28	42.75; 144.60	110.11; 109.60
C6	1	9800/9	51.41	104.91

**Table 3:** Distribution of the merged labeled drilling dataset.

Label	Material	Rows	Approx. Duration (s)	One-Second Windows
C1	Sandstone	27,392	27.391	27
C2	Shale	65,304	65.303	65
C3	Mudstone	73,400	73.399	73
C4	Bituminous coal	44,600	44.599	44
C5	Anthracite	28,000	27.999	28
C6	Lignite	8984	8.983	8

**Table 4:** Per-window torque and speed. Median and IQR are computed over the complete one-second windows; trend is Spearman ρ versus window order. The C6 trend is descriptive because only eight windows are available.

Class	Windows	Torque Median	Torque IQR	Speed Median	Torque Trend ρ
C1	27	262.82	114.42	110.97	0.482 (*p* = 0.011)
C2	65	163.24	60.45	109.90	0.915 (*p* < 0.001)
C3	73	149.50	49.82	110.36	0.716 (*p* < 0.001)
C4	44	33.35	13.00	109.05	0.294 (*p* = 0.053)
C5	28	144.60	118.11	109.60	0.628 (*p* < 0.001)
C6	8	59.19	66.64	104.86	0.976 (*n* = 8)

**Table 5:** Class-level statistics of selected sensor channels in the dataset. Values are reported as mean ± standard deviation in the corresponding acquisition units.

Label	Loading In	Loading Out	Laser Displacement	Torque	Speed
C1	4.457±0.037	2.428±0.040	7.624±0.318	283.301±116.782	110.005±6.148
C2	5.046±0.037	2.521±0.038	7.883±0.283	175.357±46.547	109.624±3.400
C3	5.081±0.036	2.669±0.039	7.777±0.315	163.572±41.824	110.234±3.701
C4	5.213±0.007	2.662±0.007	7.876±0.336	33.066±13.182	109.098±1.370
C5	4.922±0.013	2.416±0.015	7.536±0.368	111.483±68.331	109.417±3.458
C6	4.541±0.046	2.451±0.051	7.808±0.246	48.039±33.011	105.027±2.949

**Table 6:** Coal–rock identification results using the RBF-SVM classifier. Values are mean ± standard deviation across five contiguous block folds.

Input Method	Accuracy	Macro-F1
Raw features	90.26±9.26%	78.34±16.80%
WT	91.94±8.26%	80.37±17.04%
Fixed VMD–WT	92.17±7.97%	81.73±16.46%
PSO–VMD–WT	92.13±7.32%	82.17±15.70%
GWO–VMD–WT	92.13±7.32%	82.17±15.70%
IMGO–VMD–WT	92.57±6.38%	82.22±15.07%

**Table 7:** Secondary classifier results. Values are mean accuracy and macro-F1 across five contiguous block folds.

Input Method	Nearest-Centroid Acc.	Nearest-Centroid Macro-F1	Random Forest Acc.	Random Forest Macro-F1
Raw features	84.92%	78.20%	98.01%	95.47%
WT	86.32%	80.01%	99.13%	99.50%
Fixed VMD–WT	86.05%	79.70%	99.57%	99.75%
PSO–VMD–WT	86.13%	80.94%	98.39%	99.04%
GWO–VMD–WT	86.13%	80.94%	98.39%	99.04%
IMGO–VMD–WT	86.48%	80.19%	100.00%	100.00%

**Table 8:** Auxiliary feature and torque-only analyses under the same five contiguous blocks. Values are mean ± standard deviation across folds.

Analysis	Feature Set	Classifier	Accuracy	Macro-F1	Interpretation
Auxiliary	100 features	RBF-SVM	95.82±5.51%	89.98±12.98%	Exploratory extension
Auxiliary	100 features	Random forest	100.00±0.00%	100.00±0.00%	Overfitting risk
Torque-only	Torque descriptors	Nearest centroid	51.37±12.31%	43.95±11.04%	Conservative baseline
Torque-only	Torque descriptors	Random forest	58.35±12.87%	50.05±17.98%	Conservative baseline

**Table 9:** Per-class precision, recall, and F1-score computed from the aggregate confusion matrix of IMGO–VMD–WT with the RBF-SVM classifier.

Class	Support	Precision	Recall	F1-Score
C1	27	0.711	1.000	0.831
C2	65	0.969	0.969	0.969
C3	73	0.971	0.932	0.950
C4	44	0.860	0.977	0.915
C5	28	0.786	0.786	0.786
C6	8	0.500	0.500	0.500

**Table 10:** Controlled 10 dB noise-injection denoising results on measured torque windows. Values are mean ± standard deviation across 12 windows.

Method	Output SNR (dB)	SNR Gain (dB)	RMSE
Noisy input	10.00±0.00	0.00	4.104±2.896
WT	17.95±1.41	7.95	1.648±1.170
Fixed VMD–WT	19.11±0.98	9.11	1.397±0.975
PSO–VMD–WT	19.10±0.95	9.10	1.405±0.990
GWO–VMD–WT	19.12±0.93	9.12	1.404±0.980
IMGO–VMD–WT	19.19±0.89	9.19	1.379±0.953

## Data Availability

The data and analysis materials presented in this study are available on request from the corresponding author.

## Abbreviations

The following abbreviations are used in this manuscript:

IMF	Intrinsic mode function
IMGO	Improved moss growth optimization
VMD	Variational mode decomposition
WT	Wavelet thresholding
SNR	Signal-to-noise ratio
RMSE	Root mean squared error
